# Size control of precipitated particles of amino acids using a bubble column evaporator

**DOI:** 10.1016/j.heliyon.2023.e13516

**Published:** 2023-02-08

**Authors:** Atikah Wan Nafi, Mojtaba Taseidifar, Richard M. Pashley, Barry W. Ninham

**Affiliations:** a School of Science, UNSW Canberra, Northcott Drive, Canberra, ACT, 2610, Australia; b Department of Applied Mathematics, Research School of Physical Sciences, The Australian National University, Canberra, ACT, 2600, Australia

**Keywords:** Precipitation, Amino acids, Size control, Bubble column evaporator, Stirring and particle growth rates

## Abstract

The precipitation of five amino acids: DL-alanine, L-arginine, L-leucine, DL-methionine and L-tyrosine was studied at their solubility limits and isoelectric point by using a bubble column evaporator (BCE). The precipitation of amino acids via a bubble column evaporator and a standard stirring method were compared via turbidity measurements. Particle size, zeta potential and polydispersity index (PDI) were also measured using a Malvern Zeta-sizer and the particle morphology was examined using Scanning Electron Microscopy (SEM). The novel BCE process emerges as a much more effective method than precipitation by standard stirring methods. Better control of fine particle size and growth rates is achieved. The amino acids in zwitterionic form exhibit the same unexplained bubble coalescence inhibition phenomenon as do common salts. This suggests obvious applications in flotation technologies.

## 1 Introduction

The production of fine materials in the micron, sub-micron and nano-dimension ranges is of interest to many industries: ceramics, catalysis, pharmaceuticals, food, cosmetics, printing, paints, and coatings [1]. Familiar techniques for production of fine particles include spray drying, fluid grinding, fluidised bed spray, solvent evaporation, and lyophilisation [2]. Very fine particles are categorized as nanocrystals, nanocoatings, nanoparticles, and nanofibers [3,4]. Particles of nanometer size are expected to show unusual chemical and physical properties, such as high surface activity and super paramagnetism. These allow novel applications [[5], [6], [7], [8]]. Therefore, the search for novel crystallisation techniques to produce fine particles continues [[9], [10], [11], [12], [13], [14], [15]].

Precipitation induced by continuous, rapid stirring of supersaturated solutions can produce fine particles. This simplest of processes has advantages in high product yield [16]. However, considerable challenges lie in product polydispersity [17] and often, large micron-sized particles. When smaller particles do form, they often agglomerate with a broad size distribution [18,19].

This study reports an experimental examination of precipitation using a bubble column evaporator (BCE). A variety of methods for controlled precipitation exist. Temperature variation is one, and probably the first method. In Napoleon's expedition to the Nile in 1795, Claude-Louis Berthollet observed rocks of sodium carbonate on the banks of the Nile. This was contrary to expectation. Instead, calcium carbonate ought to have been precipitated from the calcium, chloride, sodium, and bicarbonate ion mixture in the flood waters [20]. The reason is that as the floods receded in the summer heat, the temperatures exceeded 50 °C. The precipitation reaction was reversed. This experiment, which marked the beginning of Physical Chemistry, has never been repeated.

Another variable that controls particle precipitation and size is water structure, which creates the local physico-chemical environment. This can be changed by the addition of indifferent solutes, ions, sugars, urea or alcohols [21]. Another parameter that affects precipitation is a strong magnetic field. One study [22] showed that temperature and a magnetic field can control the precipitation process via effects on the molecular structure of the solvent.

Similarly, a vast amount of work has gone into the manufacture of nanoparticles in reverse-micellar and microemulsion systems [23,24]. Previous studies on copper nanoparticles in anionic reverse micelles (AOT) revealed that the size of particles precipitated depended on the ratio of water to surfactant [25]. Another parameter which can affect precipitation is shaking. By this, we mean simple hand shaking, not a massive injection of energy or mechanical agitation such as by sonification, ultrasonic cavitation, vibration, and so on [26]. A remarkable study by Adam as long ago as 1948 showed that physical processes, such as shaking and the rate of bubbling, can strongly affect the growth of particles and reactivity [27]. A recent study on immunoglobulin using shaking processes discussed possible interaction of the Coulomb monopole-dipole, Archimedean forces, and the hydrophobicity effect in influencing the size of the nanobubbles produced [28].

A special issue of the journal Substantia describes many new applications of the BCE which exploits bubble coalescence inhibition by addition of salts. This includes; evaporative cooling, seawater desalination, dewatering slime, aqueous thermal inactivation, and the thermolysis of solutes [29,30].

The BCE has been used for precipitation from various salt solutions. Comparisons of the BCE method with a standard rotary stirring method for the precipitation of strontium and of calcium sulfate have been reported by us earlier [31,32]. The BCE process allowed better control of the growth of strontium sulfate and calcium sulfate particles than simple stirring.

Amino acids occur in a number of biomedical applications, with and without salts, organic solvents, and other components [[33], [34], [35], [36]]. Solubility data for amino acids [[37], [38], [39], [40]] are sparce, and little is known about amino-acid particles. Experimental production of amino acids in the last decade has been via aerosol flow reactors [[41], [42], [43], [44]]. A recent study reported that amino acids could be used as reducing agents to generate metal nanoparticle assemblies with a very useful complex nanostructure [45]. Currently, there is a paucity of methods which offer simple, cheap, and easy to set up controlled precipitation for the production of fine particles [[16], [17], [18], [19]]. This study explores the use of the BCE system to produce controlled-size particles at a laboratory scale. It suggests the potential for use of the BCE in industrial applications in future.

The BCE method shows the same effects for controlling the growth of particles of calcium sulfate, strontium sulfate (from ion pairs) and (zwitterionic) amino acids, even though they are produced from different mechanisms. Together, these studies suggest that control of particle size is a characteristic of the BCE process.

Amino acids in this study are used at their solubility limit and under solubility values. Earlier studies [31,32] of salts were used at supersaturation levels (beyond solubility). Amino acids are also found to inhibit bubble coalescence, just like common salts. Unlike salts, the amino acids are not charged, but in zwitterionic form. Remarkably the concentrations required to inhibit bubble fusion can be much lower than those for some salts.

## 2 Materials and methods

### 2.1 Materials

A series of analytical grade amino acids, DL-alanine, L-arginine, L-leucine, DL-methionine, and L-tyrosine, with purity level ≥99% (Sigma-Aldrich), were prepared at their solubility limits in Milli-Q water [33,40,46]. The samples were placed in flasks, stirred, and sonicated for 20 min, to obtain homogenous solutions. The isoelectric point (pI) and experimental solubility of each amino acid at the beginning of each experiment are listed in Table 1.

**Table 1:** pI and solubilities (mol/L) of amino-acid solutions at 25 °C before each experiment.

Solution	pI	Solubility (mol/L)
DL-alanine	6.25	1.877
L-arginine	10.8	0.854
L-leucine	6.02	0.185
DL-methionine	5.75	0.199
L-tyrosine	5.60	0.003

### 2.2 Precipitation processes

**Fig. 1:**
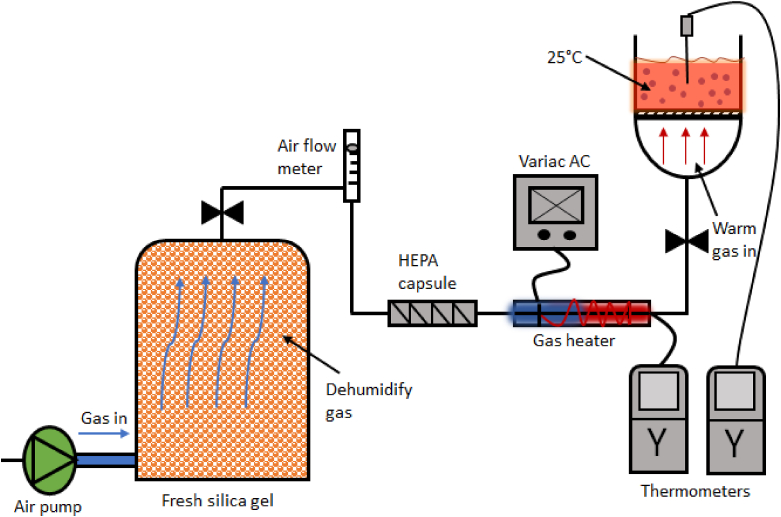
Schematic diagram of the BCE set up for precipitation of amino acids.

All of the BCE precipitation studies were carried out within a filtered air, laminar flow cabinet and around 25 °C, as shown in Fig. 1. Using the BCE system, the normal air was pumped into the amino acid solutions within the sintered column from an air pump (Hiblow HP40, Philippines). A flowmeter was placed after the desiccator to control the flow rate of the inlet air (∼10 L/min).

The inlet air was pre-filtered using a Whatman large High Efficiency Particulate Air (HEPA) filter capsule (retaining 99% of particles above 0.3 μm). Dry air (i.e. rather than cylinder N_2_) was used in these experiments because commercially this is of more practical value. The inlet temperature of air was controlled at around 70 °C to provide and maintain the temperature of the solutions within the column at around 25 °C, due the effect of evaporative cooling. The gas heater temperature was controlled by a Variac power supply and monitored using a digital thermometer (Control Company 4000 Traceable). To study the precipitation, the concentration of the amino acid solutions were used at their solubility level.

A standard rotary stirring system was used for comparison with the BCE system in the precipitation of the amino acid solutions. The standard stirring experiments on the amino acid solutions were carried out at the same concentration and temperature as for the BCE experiments. The solutions were stirred using cylindrical magnetic bars of length 3.5 cm at a stirring rate of 240 revolutions per minute (rpm). The effect of different stirring intensity (0, 120 and 480 rpm) was also studied, using L-leucine solutions at the same concentration. The characterisation and analysis of the dispersed particles of amino acids were taken from solution samples obtained directly via syringes at different times. Repeat precipitation experiments were carried out for all the amino acids listed in Table 1.

### 2.3 Analytical methods

The precipitation of the amino acids over time was measured by turbidity measurements (HACH 2100AN Turbidimeter) and by a Malvern Zetasizer (Model ZS). Clear aqueous solutions normally have lower values than 0.2 NTU (Nephelometric turbidity unit). Thus, solutions having turbidity values larger than that were considered to be at the onset of precipitation.

To determine the growth rate of the precipitated particles, 1 mL sample solutions were collected into polystyrene cells using a syringe for dynamic light-scattering (DLS) measurements using the Malvern Zetasizer (with detection size range 0.3–10 μm). The uniformity of particles was determined by their polydispersity index (PDI), given directly by the Malvern Zetasizer. The PDI ranges from 0 to 1: values close to 1 indicate a broad distribution of particles in the solution, and the sample may not be suitable for DLS measurement. The Malvern Zetasizer was also used to measure solution conductivity and the zeta potential of particles in the amino acid solutions using the BCE system. Sample solutions were filtered using 0.22 μm filter caps to remove dust and larger particles. Scanning electron microscopy (SEM; Zeiss Ultra Plus) was used for particle morphologies; after precipitation, the amino-acid solutions were filtered through 0.22 μm membranes. They were kept in a filtered laminar airflow cabinet to complete drying before SEM examination.

## 3 Results and discussion

### 3.1 Precipitation of amino acids in BCE

The ionization of amino acids, used as in this work, is given by the reactions in Eq. (1) and Eq. (2):

(1) $$R-CH\left({NH}_{3}^{+}\right)-{COO}^{-}+H^{+}\to R-CH\left({NH}_{3}^{+}\right)-COOH$$

(2) $$R-CH\left({NH}_{3}^{+}\right)-{COO}^{-}+{OH}^{-}\to \;R-CH\left({NH}_{2}\right)-{COO}^{-}+H_{2}O$$

The precipitated particles of amino acids and their morphology were compared using SEM.

Air was passed through a gas heater to provide a supply of stable, warm, dry bubbles in the column containing the amino acid solutions; water vapour was continuously removed from the top of the BCE. This evaporation increased the concentration or saturation level of the solutions slowly, and so would be expected to enhance precipitation compared to simple stirring. The BCE process has two main controlling variables: the flow rate of the dry air and the temperature of the inlet gas, both of which determine the water evaporation rate. However, previous studies on inorganic precipitates have demonstrated a surprising effect: the high density of bubbles present in the BCE inhibits the growth of large particles, even in supersaturated solutions, and acts to favour small particles [31,32]. The BCE method was found to be able to precipitate fine particles over the range from micron-to nano-size in a controlled manner.

**Fig. 2:**
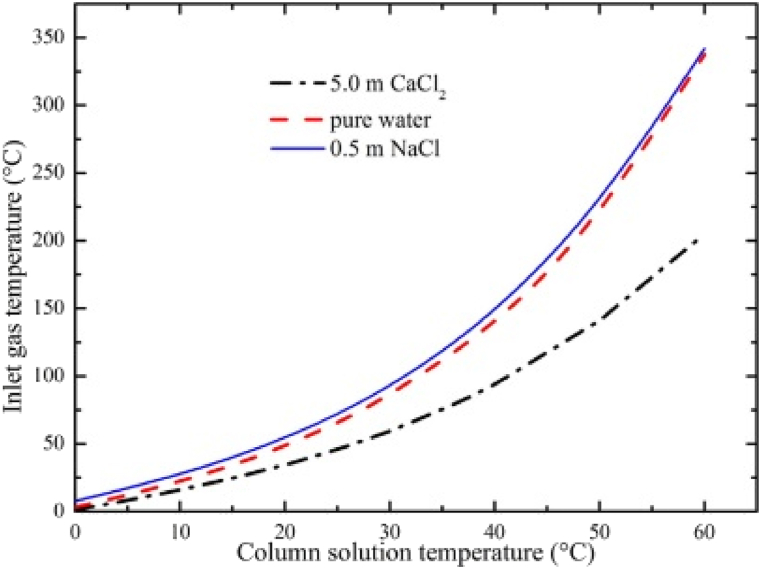
Relationship between temperature of inlet-gas and temperature of column-solution at steady state in the BCE system [47].

Fig. 2 shows the relationship between the temperature of inlet-gas and the temperature of solution within the column at thermal steady state; this was independent of the inlet-gas flow rate. For example, based on Fig. 2, the solution temperature inside the BCE in this study was maintained at 25^ᵒ^C by using an inlet gas temperature at around 50^ᵒ^C. Normally, once particles start to precipitate, the solute concentration decreases. Precipitation stops once the solubility limit is reached. However, in the BCE process, the concentration of the solute should increase because of the continuous water-vapour loss; and this should favour particle growth.

In the BCE, the evaporation rate can be approximated by Eq. (3):

(3) $$r_{c}=r_{f}\;\frac{T_{c}/T_{f}}{P_{c}/P_{f}}\;\frac{\rho_{v}^{w}}{\rho^{w}}$$

where *r*_*c*_ is the evaporation rate (the volume of evaporated water lost per unit time assuming 0% humidity of the dry inlet gas and only water is vaporised from the solution) and *r*_*f*_ the flow rate of the inlet gas. *T*_*c,*_ the temperatures of the gas at the top of the column and *T*_*f*_, temperature of inlet gas. *P*_*c*_ and *P*_*f*_ are the corresponding pressures at the same positions. $\rho_{v}^{w}$ is the water-vapour density (mol/m^3^) of the amino acid solutions at temperature *T*_*c*_ and *ρ*^*w*^ the density of pure liquid water (mol/m^3^), also at *T*_*c*_ [48].

The concentration of solution in the BCE after the precipitation process can be obtained from the volume of water vaporised from the solution (Eq. (4)):

(4) $$C_{t}=\frac{C_{i}V_{i}}{V_{i}-r_{c}t}$$

where *C*_*t*_ is the concentration of amino-acid solution at time *t*, *C*_*i*_ the initial concentration, and *V*_*i*_ the initial volume of the amino acid solutions*.*

### 3.2 Effects of amino-acid zwitterionic solutes on bubble-coalescence inhibition

**Fig. 3:**
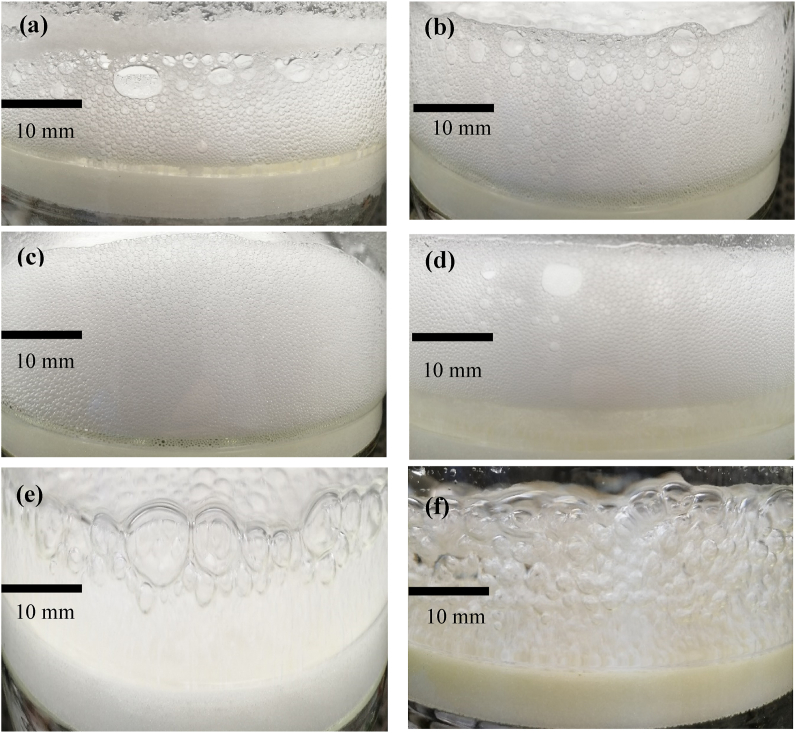
Photograph of the amino-acid solution of (a) DL-alanine, (b) L-arginine, (c) L-leucine, (d) DL-methionine, (e) L-tyrosine, and (f) Milli-Q water, after 180 min in the BCE column.

Amino acids are typically zwitterionic in the pH range 4–10. Amino acids have fairly low water solubility and almost neutral particle surface charge. Under acidic conditions (pH < 4) or basic conditions (pH > 10), solutions of amino acids are soluble due to the dominant ionic form of either the protonated amine or the ionised carboxylic acid. Bubble sparging into the amino acid solutions (with the exception of L-tyrosine) produced very small bubble foam, less than 1 mm in diameter, compared to bubble sparging into Milli-Q water (Fig. 3). Tyrosine showed very small bubbles at the sinter, but large bubbles formed by coalescence at the top of the solution. This indicates that inhibition of bubble coalescence occurred for the amino acids, as observed with many common salts. The fine bubbles also appear to aid in the control mechanism to produce finer particles.

To test if the precipitation of the amino acids caused this bubble-coalescence inhibition, solutions of L-leucine were studied at 0.1 M, well below its solubility level (at pH 6). The bubble coalescence inhibition was also observed in the more-dilute, non-saturated solutions, and showed quite similar behaviour to NaCl solutions of the same molarity.

Amino acids can be considered to be very weak surfactants and, when the gas flow was stopped, the foam collapsed, as expected. The foam was clearly formed by the combination of continuous gas flow and strong bubble-coalescence inhibition.

The observation that zwitterionic amino acid solutions behave like many salts in preventing bubble coalescence at similar concentrations suggests that the inhibition mechanism cannot be related to selective ion adsorption at the air-water interface [49]. Some neutral solutes, such as sucrose, have a less significant effect on bubble coalescence inhibition at concentration of 0.15 M [50,51]. Hence, it appears that ionic and zwitterionic solutions are the most effective non-surfactant solutes for coalescence inhibition.

Further recent studies on bubble coalescence in amino acid solutions have examined and discussed possible mechanisms and whether it might be related to dipole effects at the air-water interface [52].

### 3.3 Amino-acid precipitation in the BCE and the standard rotary stirring method

**Fig. 4:**
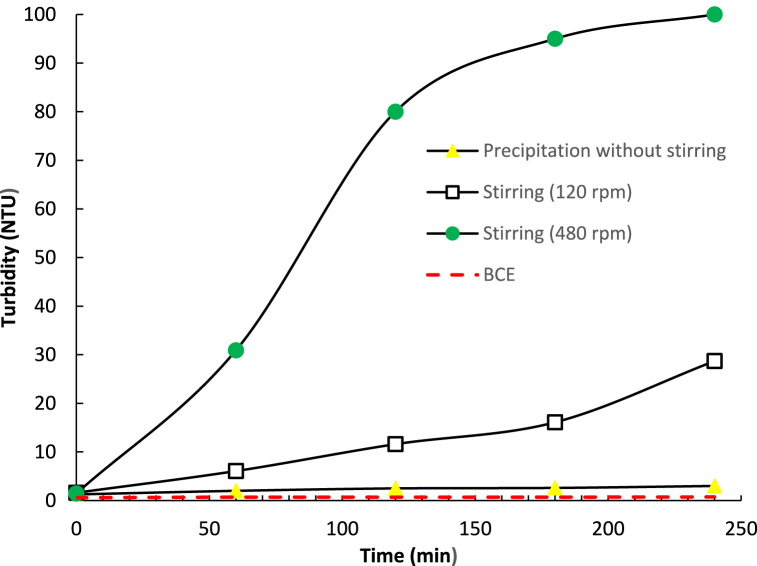
Precipitation of L-leucine, monitored by turbidity measurements, in the standard stirring process at different stirring speeds, and in the BCE process.

**Fig. 5:**
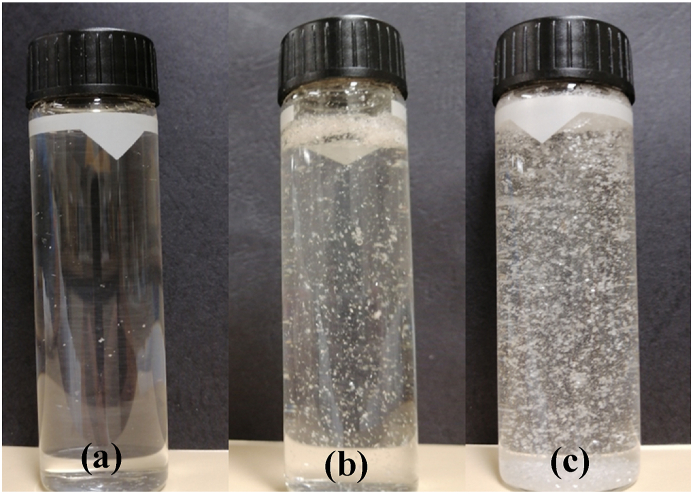
Photograph of L-leucine precipitation in stirring method at speed intensity of (a) 0 rpm, (b) 120 rpm, and (c) 480 rpm.

**Fig. 6:**
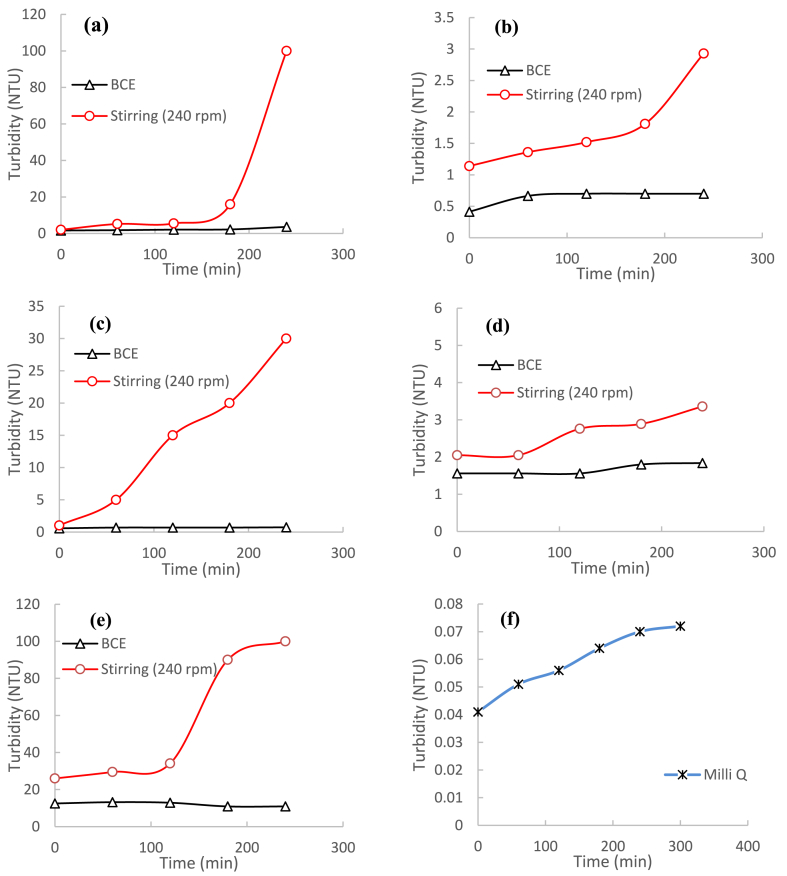
Precipitation of amino acids, monitored by turbidity measurements: (a) DL-alanine, (b) L-arginine, (c) L-leucine, (d) DL-methionine, (e) L-tyrosine, and (f) Milli-Q water in the BCE and in the standard stirring method at 240 rpm.

Fig. 4 shows the particle growth rate of L-leucine via solution turbidity measurements in the BCE process. This is compared with a standard rotary experiment with different rotary speeds. Fig. 5 shows a photograph of the L-leucine solution after 240 min with different stirring rates. Fig. 6 shows particle growth in the precipitation process of five amino acids in the standard stirring system (240 rpm) and in the BCE. The turbidity of all the amino-acid solutions in both systems showed the onset of precipitation (turbidity >0.2) from the beginning of the experiment.

During the precipitation process, particle growth was monitored by turbidity. An increase in the turbidity correlated with particles of size larger than 5 μm [53]. The turbidity of L-leucine in the standard stirring rotary method at 480 rpm increased rapidly from the beginning of the experiment, while the turbidity of the solution without stirring showed similar behaviour to the solution vigorously stirred in the BCE process.

These observations show that a higher rate of stirring increased the precipitation of L-leucine (Fig. 5). In contrast, all the amino acid solutions in BCE method gave constant turbidity over more than 200 min (Fig. 4, Fig. 6), even though the amino acid solutions used in both systems were at the same concentration. In the BCE system, the cloudiness of the quiescent amino acid solutions cleared, with low turbidity throughout the experiment. The turbidity of all amino-acid solutions in the standard stirring system increased rapidly with obvious white precipitated particles. The differences, compared to the same sample solution in the BCE system, were quite marked.

It might have been expected that the continuous water evaporation in the BCE would favour particle growth because of the increasing amino acid concentration. However, the BCE system instead inhibited particle growth. The particle growth in the BCE was possibly disturbed by the complex behaviour of the bubbles, giving a quite different effect to that produced by increasing the stirring rate. This was also found in earlier studies using SrSO_4_ and CaSO_4_ solutions [31,32]. Those initial studies suggest that controlled precipitation and the production of fine particles could be a characteristic of the BCE.

### 3.4 Average size and the polydispersity index (PDI) of amino-acid solutions

**Fig. 7:**
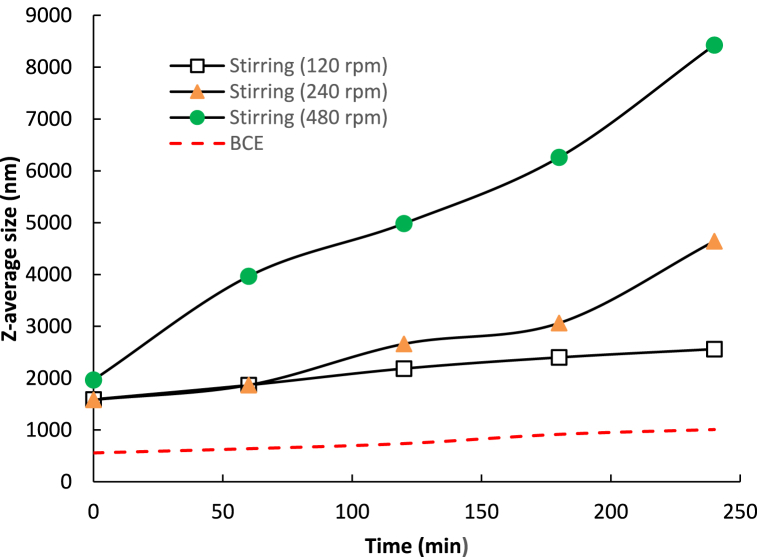
Average size of L-leucine precipitates in the standard stirring process at different stirring speeds, and in the BCE process, both at around 25 °C.

**Fig. 8:**
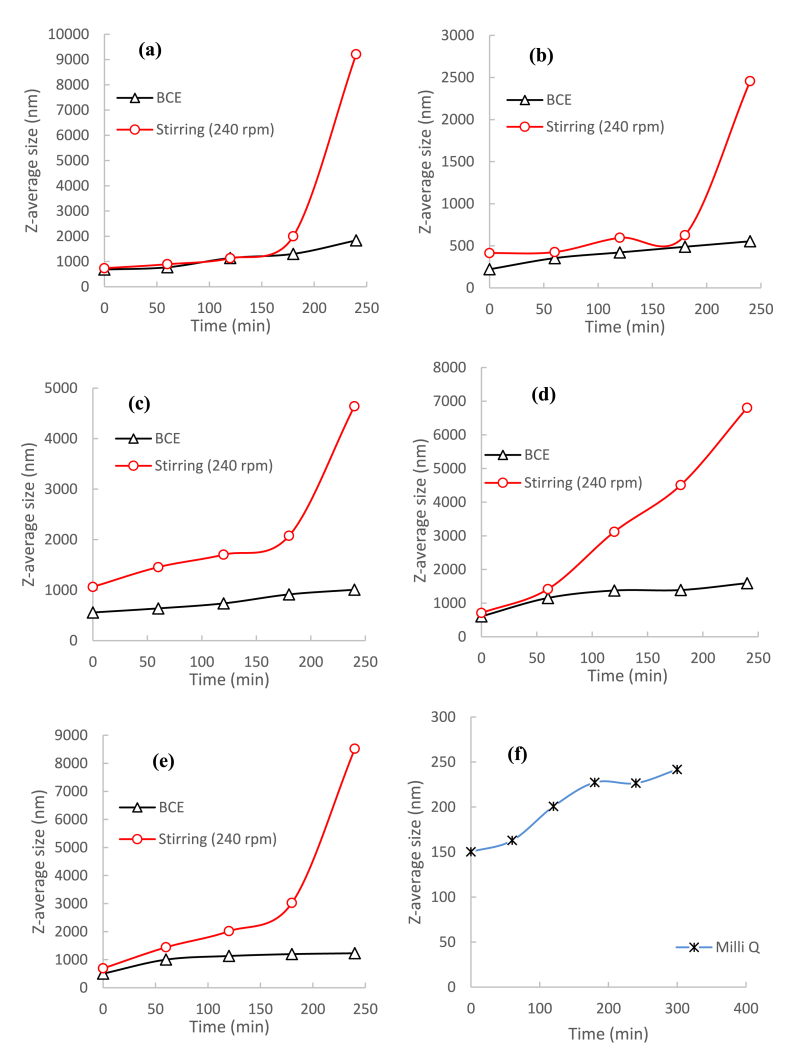
Precipitation of amino acids monitored by average size: (a) DL-alanine, (b) L-arginine, (c) L-leucine, (d) DL-methionine, (e) L-tyrosine, and (f) Milli-Q water in the BCE and in the standard stirring method at 240 rpm.

A limitation of the turbidimeter measurement means that it is not able to detect very small particles [54,55]. Hence, the growth of L-leucine and the other amino acids particles in the BCE and standard rotary stirring method was also measured using a Malvern zeta-sizer. The average size changes observed are shown in Fig. 7, Fig. 8.

The average particle size/diameter in all the amino acid solutions initially was around 1 μm, consistent with the onset precipitation measured by turbidity. In the BCE system, this increased slightly over time, but remained below 2 μm. In contrast, the average particle size in all the amino acid solutions in the standard stirring method increased rapidly.

**Fig. 9:**
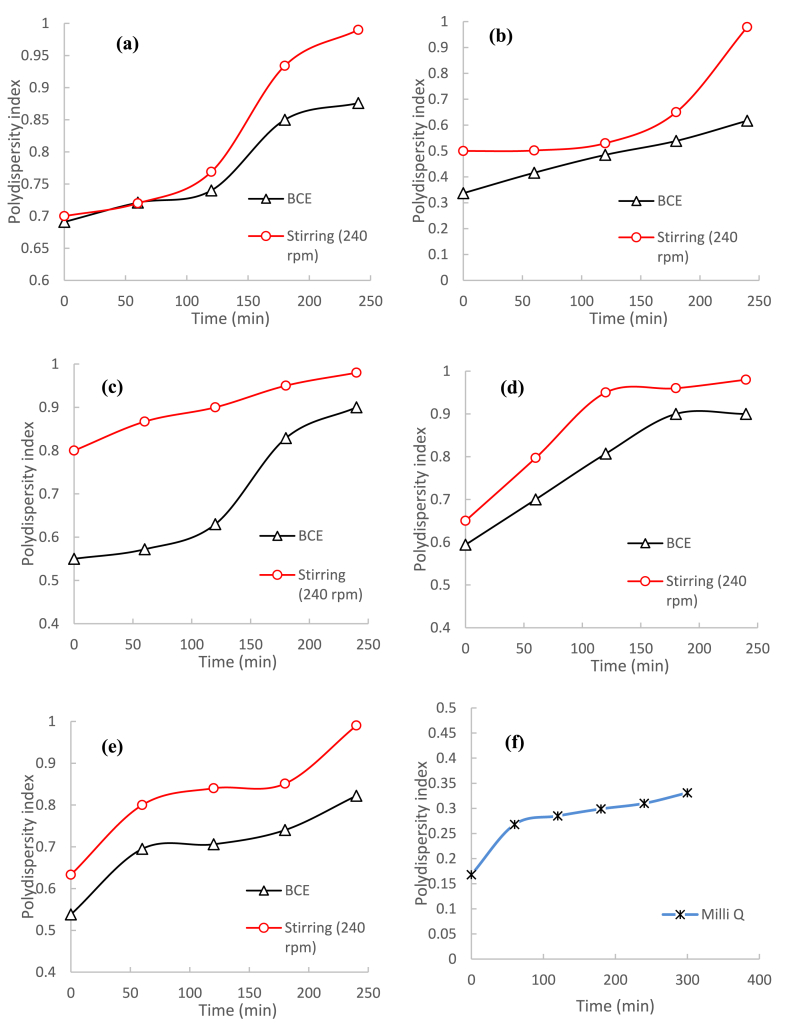
Precipitation of amino acids monitored by Polydispersity index (PDI): (a) DL-alanine, (b) L-arginine, (c) L-leucine, (d) DL-methionine, (e) L-tyrosine, and (f) Milli-Q water, in the BCE and in the standard stirring method at 240 rpm, all at around 25 °C.

Fig. 9 shows the polydispersity index (PDI) in the amino acid solutions over time measured using the Malvern Zetasizer. A PDI was used as a measurement to the uniformity of precipitated particles in the solutions, with values close to 1 indicating the formation of a broad range of particle sizes. The particles from the BCE system were significantly more uniform in size compared to those precipitated in the standard stirring system. The amino acids precipitates in the standard stirring system not only experienced more rapid growth but also had higher PDI values from the initial point and a broader size distribution. At the end of the precipitation time, the PDI of all amino acids approached 1 in the standard stirring system but showed only a gradual increase in the BCE system.

### 3.5 Particle morphology

**Fig. 10:**
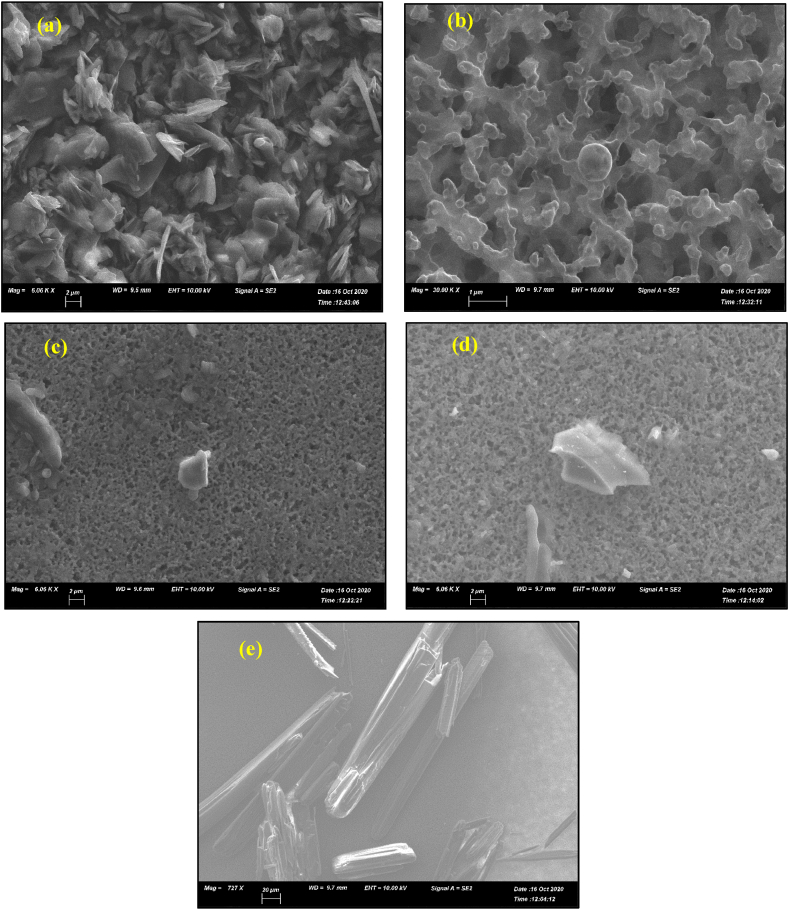
SEM images of the different amino-acid: (a) DL-alanine, (b) L-arginine, (c) L-leucine, (d) DL-methionine, and (e) L-tyrosine precipitated in the BCE system after 200 min.

Fig. 10 shows typical SEM images of precipitated particles for all five amino acids in the BCE method. The particle morphology of L-arginine, L-leucine and DL-methionine showed small fine particles of size around 600 nm to 2 μm. L-arginine formed spherical particles. L-tyrosine produced very small amounts of rod-shaped or pallet-like particles. DL-alanine produced flake shapes of uniform size. The SEM results reinforce the conclusion that the inhibition effect of particle growth in the BCE, has the potential for use as a novel process to produce particles with desired sizes in a controlled manner.

### 3.6 Conductivity and zeta potential

The precipitation process in the BCE system was also monitored using zeta-potential measurements. Table 2 shows the results obtained for each amino acid. After 180 min of precipitation of the five amino acid solutions in the BCE system, there was a slight increase in the zeta potential of the particles. The pH of the solutions remained close to their respective isoelectric points. The electrical conductivities of each solution were low because of the zwitterionic nature of these solutes at natural pH, which is around the isoelectric point (pI). The surface charge densities on the precipitated particles were also very low in magnitude, given the low negative zeta potentials observed and the relatively long Debye lengths of these solutions. It has been reported [56] that parameters such as molecular weight, concentration and isoelectric point (pI) value affect the conductivities and Zeta potentials. These values differ significantly, for the different amino acids.

**Table 2:** Conductivity, pH and zeta potential of amino acid solutions in the BCE.

Solution	Time (min)	0	30	60	90	120	150	180
DL-alanine	pH	6.25	6.26	6.29	6.30	6.32	6.35	6.30
	Conductivity (mS/cm)	0.44	0.79	0.90	0.98	1.06	1.10	1.12
	Zeta potential (mV)	−16.7	−7.06	−5.63	−5.18	−1.96	−0.85	−12.5
L-arginine	pH	10.78	10.75	10.78	10.82	10.86	10.90	10.94
	Conductivity (mS/cm)	0.49	0.91	1.13	1.46	1.84	2.15	2.54
	Zeta potential (mV)	−2.12	−1.67	−1.47	−1.53	−1.15	−0.65	−0.87
L-leucine	pH	5.98	6.11	6.15	6.21	6.20	6.20	5.98
	Conductivity (mS/cm)	0.01	0.02	0.02	0.02	0.02	0.03	0.03
	Zeta potential (mV)	−0.64	−0.36	−0.26	−0.19	−0.14	−0.04	−0.03
DL-methionine	pH	5.74	5.75	5.81	5.86	5.91	5.93	5.88
	Conductivity (mS/cm)	0.01	0.02	0.02	0.03	0.03	0.04	0.04
	Zeta potential (mV)	−1.32	−0.68	−0.67	−0.35	−0.18	−0.16	−0.13
L-tyrosine	pH	5.60	5.72	5.79	5.84	5.87	5.98	5.92
	Conductivity (mS/cm)	0.003	0.02	0.03	0.03	0.03	0.04	0.04
	Zeta potential (mV)	−0.79	−0.52	−0.42	−0.39	−0.36	−0.23	−0.24

The BCE method shows remarkable effectiveness in controlling the growth of amino acids unlike the stirring method; and this has been tested for several amino acids in this current work. In addition, amino acids, as for electrolytes [50,51], inhibit bubble coalescence. Unlike salts, amino acids are zwitterionic. An explanation remains an open question.

**Fig. 11:**
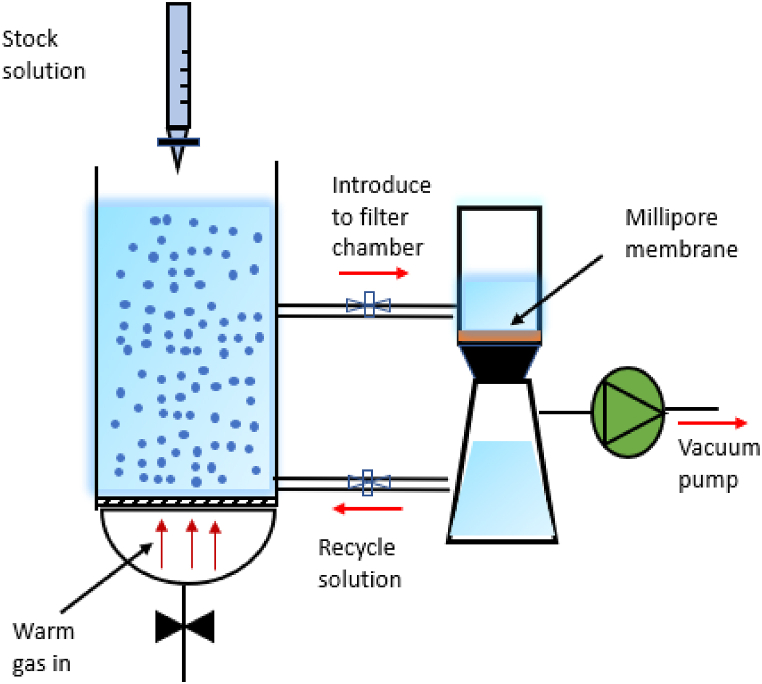
Pilot plant diagram to produce particles using BCE.

The particle yield was not measured in this work, because the overall precipitation levels of the amino-acid solutions in the BCE process were quite low. It would be possible to increase the yield by continuously removing the fine particles, using, for example, a membrane nano-filtration system (Fig. 11). This study was focused on producing the desired particle size achieved using the BCE column so that the product could be collected after being filtered through suitable membranes using a vacuum pump. Then the filtered solution can be recycled to the BCE column and its concentration can be adjusted using a stock solution.

Extensive efforts have been made to develop better fine particle manufacturing technologies, to make new materials more viable and cost effective. For example, biosurfactants derived from amino acids, have gained attention in various industries such as in pharmaceutical, petroleum, food industries and so on. Despite this increasing demand, this is still at an early phase of production [[57], [58], [59]]. Precipitation using the BCE method offers a low cost, energy wise, simple and high reusability process; offering the possibility to address this production limitation with improved particle-controlled properties.

## 4 Conclusions

By comparison to the standard rotary stirring method, the BCE process has an inhibitory effect on the precipitation induction time and the precipitate growth rate. This should allow controlled production of amino-acid particles over a wide size range, from nanometer to micrometer range. Our results are similar to previous studies on the precipitation of salt solutions at supersaturated conditions. Together these studies suggest that precipitation control is a general property of bubble column evaporators. The technique should be useful for the production of fine particles for industrial applications in ceramics, catalysis, cosmetics, pharmaceuticals, and in food products. Zwitterionic amino acids may also be used in froth flotation and ion-flotation processes to reduce bubble size and improve efficiency without the addition of added salt. This might be an advantage in, for example, using amino acids with or without surfactant aid to replace PFAS in fire-fighting foams; and also as a green adsorbent for the removal of PFAS contaminants.

## Credit author statement

Atikah Wan Nafi, Mojtaba Taseidifar: Conceived and designed the experiments; Performed the experiments; Analyzed and interpreted the data; Contributed reagents, materials, analysis tools or data; Wrote the paper.Richard Mark Pashley, Barry. W. Ninham: Conceived and designed the experiments; Analyzed and interpreted the data; Contributed reagents, materials, analysis tools or data; Wrote the paper.

## Funding statement

This research did not receive any specific grant from funding agencies in the public, commercial, or not-for-profit sectors.

### Data availability statement

Data included in article/supp. Material/referenced in article.

## Declaration of competing interest

The authors declare that they have no known competing financial interests or personal relationships that could have appeared to influence the work reported in this paper.
